# Supplementary material for: Deletion of wfs1 Impairs Oligodendrocyte Precursor Cells Dorsal Distribution and Myelination Through the wfs1-hmgcs1 Axis in Zebrafish
Source: Biology (Basel). 2026 Aug 16;15(16):1402. doi: 10.3390/biology15161402 (PMC13510151; doi:10.3390/biology15161402)
Supplement: Supplementary file 1 [file biology-15-01402-s001.zip › File S1-Transcriptome sequencing data and differentially expressed genes (DEGs) in wfs1-- zebrafish at 5 dpf/2.KEGG/wfs1KOvsWT/ALL/src/dre04142.html]

dre04142


- dre:569298

- Up regulated genes

569298(1.32871616109562)

- dre:450083

- Up regulated genes

450083(2.11033262833863)

- dre:567720

- Up regulated genes

567720(3.89164392565199)

- dre:566517

- Up regulated genes

566517(1.38398827270854)

- dre:393657

- Up regulated genes

393657(3.3043946758273)

- dre:100003043

- Up regulated genes

100003043\_1(5.33116958051084)

- dre:571066

- Down regulated genes

571066(-7.1936807901335)

- dre:368911

- Up regulated genes

368911(2.8226812494078)

- dre:570148

- Up regulated genes

570148\_1(1.99799176335957)

- dre:565099

- Up regulated genes

565099(2.29036294338696)

- dre:406511

- Up regulated genes

406511\_1(1.02537112230584)

- Down regulated genes

406511(-1.07481122268925)

- dre:565246

- Down regulated genes

565246(-2.15380681025991)

- dre:337771

- Down regulated genes

337771(-1.26044614136241)
- dre:445244

- Up regulated genes

445244(2.2381033770278)

- Down regulated genes

445244\_1(-1.27471708253071)
- dre:327237

- Up regulated genes

327237\_1(2.47898625223781)

- dre:337771

- Down regulated genes

337771(-1.26044614136241)
- dre:445244

- Up regulated genes

445244(2.2381033770278)

- Down regulated genes

445244\_1(-1.27471708253071)
- dre:327237

- Up regulated genes

327237\_1(2.47898625223781)

- dre:436725

- Up regulated genes

436725\_1(6.01413573574431)

- Down regulated genes

436725(-2.29340566555652)

- dre:553423

- Up regulated genes

553423(1.20206657950112)

- Down regulated genes

553423\_1(-1.89310687503167)

- dre:559575

- Up regulated genes

559575(1.98928752692638)

Close
